# Allosteric modulatory effects of SRI-20041 and SRI-30827 on cocaine and HIV-1 Tat protein binding to human dopamine transporter

**DOI:** 10.1038/s41598-017-03771-0

**Published:** 2017-06-16

**Authors:** Wei-Lun Sun, Pamela M. Quizon, Yaxia Yuan, Wei Zhang, Subramaniam Ananthan, Chang-Guo Zhan, Jun Zhu

**Affiliations:** 1 Department of Drug Discovery and Biomedical Sciences, College of Pharmacy, University of South Carolina, Columbia, SC USA; 2 Molecular Modeling and Biopharmaceutical Center, University of Kentucky, Lexington, KY USA; 3 Center for Pharmaceutical Research and Innovation, University of Kentucky, Lexington, KY USA; 4 Department of Pharmaceutical Sciences, College of Pharmacy, University of Kentucky, Lexington, KY USA; 5 Department of Chemistry, Drug Discovery Division, Southern Research Institute, Birmingham, AL USA

## Abstract

Dopamine transporter (DAT) is the target of cocaine and HIV-1 transactivator of transcription (Tat) protein. Identifying allosteric modulatory molecules with potential attenuation of cocaine and Tat binding to DAT are of great scientific and clinical interest. We demonstrated that tyrosine 470 and 88 act as functional recognition residues in human DAT (hDAT) for Tat-induced inhibition of DA transport and transporter conformational transitions. Here we investigated the allosteric modulatory effects of two allosteric ligands, SRI-20041 and SRI-30827 on cocaine binding on wild type (WT) hDAT, Y470 H and ﻿Y﻿88 F mutants. Effect of SRI-30827 on Tat-induced inhibition of [^3^H]WIN35,428 binding was also determined. Compared to a competitive DAT inhibitor indatraline, both SRI-compounds displayed a similar decrease (30%) in IC_50_ for inhibition of [^3^H]DA uptake by cocaine in WT hDAT. The addition of SRI-20041 or SRI-30827 following cocaine slowed the dissociation rate of [^3^H]WIN35,428 binding in WT hDAT relative to cocaine alone. Moreover, Y470H and Y88F hDAT potentiate the inhibitory effect of cocaine on DA uptake and attenuate the effects of SRI-compounds on cocaine-mediated dissociation rate. SRI-30827 attenuated Tat-induced inhibition of [^3^H]WIN35,428 binding. These observations demonstrate that tyrosine 470 and 88 are critical for allosteric modulatory effects of SRI-compounds on the interaction of cocaine with hDAT.

## Introduction

Despite the widespread use of efficacious antiretroviral therapies to control peripheral human immunodeficiency virus (HIV) infection and improve the life of HIV patients, HIV-associated neurocognitive disorders (HAND) remain highly prevalent and represent a significant health problem^1^. It is commonly accepted that viral replication and proteins within the central nervous system (CNS) play a central role in the development of HAND^2^ particularly since most Highly Active Antiretroviral Therapy (HAART) medications do not cross the blood-brain barrier, while infected macrophages carrying the virus can^3^. Dopamine (DA) is essential for a variety of brain activities involved in attention, learning, memory^4, 5^, and motivation^6, 7^. Converging lines of clinical observation, supported by imaging^8, 9^, neuropsychological performance testing^10, 11^, and postmortem examinations^12^, have implicated DA dysregulation with the abnormal neurocognitive function observed in HAND^13, 14^. DA-rich brain regions are highly susceptible to the effects of both HIV infection and substance use. In the early stage of HIV infection, increased levels of DA and decreased DA turnover are found in the cerebrospinal fluid of therapy-naïve HIV patients in asymptomatic infection^15^, which may contribute to decreased levels of DA in DA-rich brain regions in the advanced stages of HIV infection^11, 16, 17^. Importantly, HIV-induced elevated levels of extracellular DA in CNS can stimulate viral replication in human macrophages within DA-rich brain regions^2, 18, 19^, further resulting in viral protein release, which has been implicated in the pathophysiology of HAND^20^. Cocaine abuse has been shown to increase the incidence of HAND and exacerbate the severity of HAND by enhancing viral replication^21–27^. Currently, there are no promising therapeutic approaches for cocaine addiction and HIV infection associated comorbidities^28^. Therefore, there is a pressing need to define the molecular mechanism(s) by which the impaired dopaminergic system by HIV-1 infection affects the progression of HAND in concurrent cocaine abusers.

The presynaptic dopamine transporter (DAT) plays an essential role in dopamine homeostasis and maintaining stable synaptic dopaminergic tone involved in attention, learning, memory^4, 5^, and motivation^6, 7^. Cocaine acts as a non-translocated inhibitor and exhibits non-selective binding to the DAT, serotonin transporter and norepinephrine transporter. However, the strong psychoactive behavioral responses and addictive effects of cocaine are mediated almost exclusively by its interaction with the DAT^29, 30^. DAT is a primary target for cocaine binding, which has been shown to overlap DA uptake site^31^. In addition to competitive inhibitors and substrates of DAT, there is growing interest in allosteric modulation of DAT. Allosteric sites on human DAT (hDAT) may represent novel drug targets that display neutral cooperativity with the classical DA uptake site. There are a number of advantages in using allosteric modulators of DAT as preferred therapeutic agents over classic competitor of the DA uptake site with minimal effects on the basal DA transmission but decreasing the cocaine’s action on DAT. For example, it has been shown that allosteric modulators of DAT such as the SRI-compounds act as partial antagonists of DA uptake without the full inhibitory profile that is typical of classic competitors of DAT^32–34^. In rat synaptosomes, SRI-compounds diminish cocaine’s ability to inhibit DA uptake^35^, however, their effect on the interaction between cocaine and hDAT is still unknown. Further, it is uncertain whether the SRI-compounds suppressive effect on cocaine inhibition of DA uptake is mediated through their interaction with DAT, since these compounds also partially inhibit both serotonin and norepinephrine transporters^36, 37^.

HIV-1 viral proteins are associated with the persistence of HIV-related neuropathology and subsequent neurocognitive deficits^38–41^. Among viral proteins, Tat protein plays a crucial role in the neurotoxicity and cognitive impairment evident in neuroAIDS^42, 43^. DAT activity is strikingly reduced in HIV-1-infected cocaine-using patients, correlating with the severity of HIV-1 associated cognitive deficits^8, 9^. We have demonstrated that Tat directly binds to DAT^44, 45^. Exposure to Tat alone results in an inhibition of DA transport and promotes the internalization of DAT^44, 46, 47^. Interplay of Tat and cocaine augments synaptic DA levels and Tat release by inhibiting DAT activity^45, 48^, which may contribute to the progression of HAND underlying the cognitive deficits in HIV-1 positive cocaine-using individuals^8, 9^. Similarly, conditioned expression of Tat in the mouse brain further potentiates cocaine rewarding *in vivo* ^49^. Together, these results suggest a synergistic effect of cocaine and Tat on DA transmission contributing to cognitive dysfunction and elevating the cocaine additive effects.

**Figure 1:**
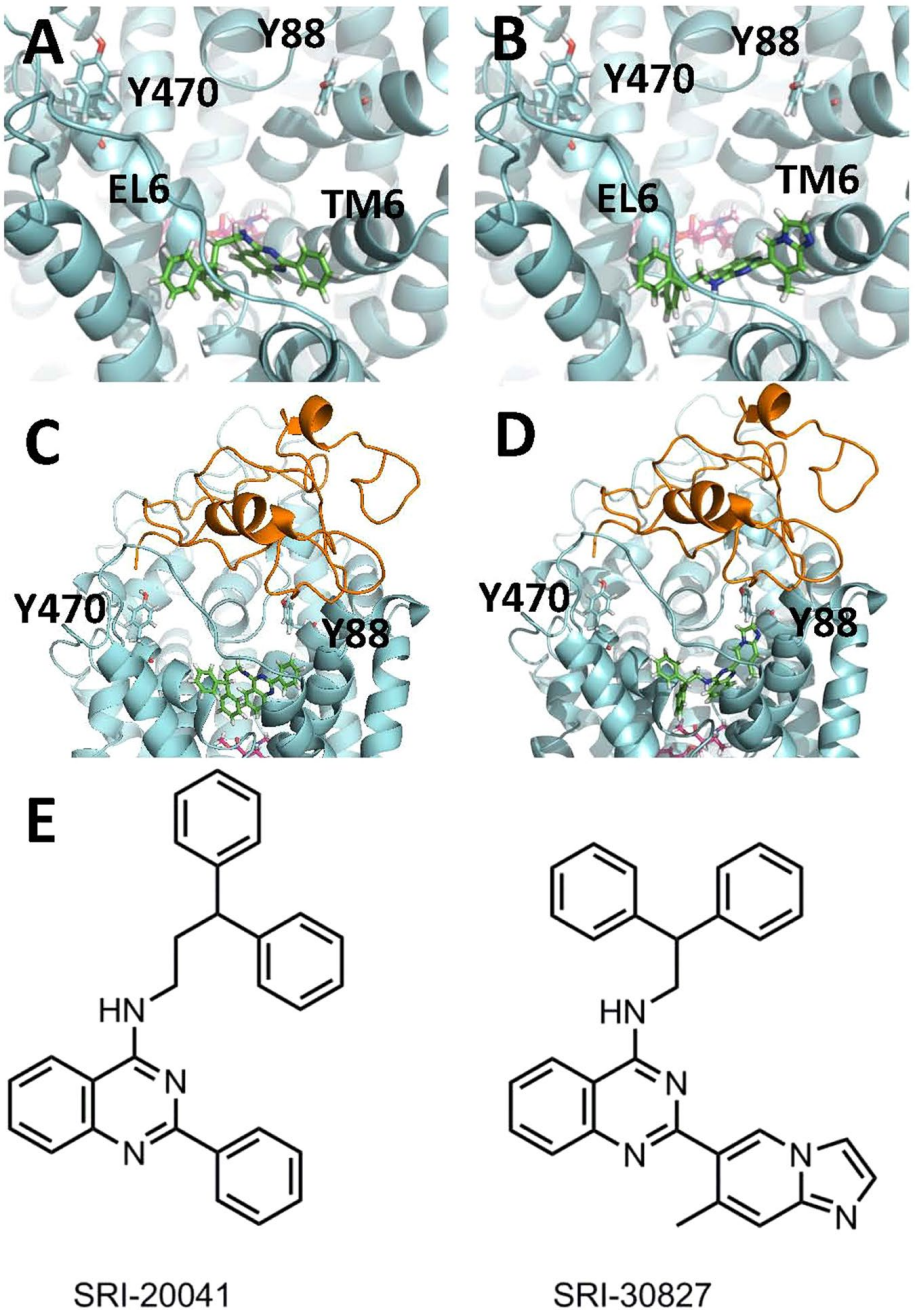
Molecular modeling of binding sites of SRI-20041 and SRI-30827 on DAT. (**A**) The cartoon presentation of the homology model of hDAT^66^ (based on the crystal structure of dDAT, PDB ID: 4M48) with SRI-20041 docked to allosteric binding site on the extracellular vestibule. Key residues Tyr470 and Tyr88 are shown in stick style. SRI-20041 and cocaine are shown in green-colored and red-colored solid-sticks, respectively. (**B**) The cartoon presentation of the homology model of hDAT with SRI-30827 docked to the allosteric binding site on the extracellular vestibule. SRI-30827 and cocaine are shown in green-colored and red-colored solid-sticks, respectively; (**C**) and (**D**) SRI-20041 and SRI-30827 were docked to the Tat-hDAT model^66^, respectively. Tat protein is shown in orange-colored cartoon (**E**) structure of the two allosteric ligands, SRI-20041 and SRI-30827.

Our previous studies showed that Tat displays an allosteric modulatory effect on DAT function^35, 45^. Through integrated computational modeling prediction and experimental validation, we explored structural regions of Tat-hDAT model to identify potential binding sites for Tat^50^. The structure of human DAT was homology modeled based on the X-ray crystal structure of drosophila DAT (dDAT)^51^ in our previous studies^47, 50, 52^. The sequence identity between hDAT and dDAT is 46%, which is considered to be sufficient for constructing a satisfactory homology model^53, 54^. We identified that DAT tyrosine 470 and 88 replaced by histidine (Y470H) or phenylalanine (Y88F) retained the normal surface DAT expression and inhibited Tat-induced inhibition of DA transport^44, 47^. Further, mutating these two residues prevented zinc-induced conformational transporter transitions^44, 47^, which may suggest that these two residues are critical for Tat allosteric modulation of DAT. In this study we first evaluated the effects of SRI-20041 and SRI-30827 (see Fig. 1), novel allosteric modulators of DAT^32^, on cocaine-mediated inhibitory effect on hDAT function. Further, we assessed whether the tyrosine 470 and 88 are critical for the allosteric modulation of hDAT by the SRI-compounds in modulating cocaine’s interaction with DAT. Finally, we determined whether SRI-compounds attenuates Tat-induced inhibition of DAT binding sites. Understanding the allosteric sites of DAT may uncover new therapeutic avenues for treating both cocaine addiction and cocaine-accelerated HAND in HIV-1 positive individuals.

## Results

### Mutations of Y470 and Y88 attenuate effects of SRI-20041 on cocaine-induced inhibition of DAT activity

To determine whether Y470 and Y88 are responsible for the SRI-20041-mediated allosteric modulation of hDAT, we first assessed inhibition of [^3^H]DA uptake by cocaine in the presence of SRI-20041 or indatraline. As shown in Table 1, Two-way ANOVA on the apparent affinity (IC_50_) for cocaine inhibiting [^3^H]DA uptake revealed significant main effects of drug (F_(2,56)_ = 13.71, *p* < 0.001) and mutation (F_(3,56)_ = 37.53, *p* < 0.001), respectively. Post hoc analysis indicated that cocaine IC_50_ values were significantly decreased in Y470H-hDAT (196 ± 23 nM, LSD: *p* < 0.001) and Y88F-hDAT (157 ± 10 nM, *p* < 0.001) but not in Y470F-hDAT (281 ± 16 nM), compared to the WT-hDAT (324 ± 19 nM). These results are consistent with our previous studies^44, 47^, suggesting that the mutations of Y470 and Y88 increase cocaine-mediated inhibition of DA uptake. The application of SRI-20041 (12.8 µM), an allosteric modulator, significantly increased the cocaine IC_50_ values of [^3^H]DA uptake ~35% in WT-hDAT (324 ± 19 to 431 ± 32 nM, *p* < 0.05) and ~40% in Y470F-hDAT (281 ± 16 to 394 ± 34 nM, *p* < 0.05), respectively. In contrast, SRI-20041 did not alter the inhibition of [^3^H]DA uptake by cocaine in either Y470H-hDAT (203 ± 28 nM) or Y88F-hDAT (156 ± 18 nM), indicating that mutations of Y470 and Y88 attenuate the allosteric modulatory effect of SRI-20041 on DA uptake. Indatraline (10 nM), a control drug for competitive inhibition of DA uptake, significantly increased IC_50_ values of cocaine from 324 ± 19 to 424 ± 23 nM in WT-hDAT (*p* < 0.05), from 196 ± 23 to 301 ± 38 nM in Y470H-hDAT (*p* < 0.05), from 281 ± 16 to 371 ± 32 nM in Y470F-hDAT (*p* < 0.05), and from 157 ± 10 to 256 ± 31 nM in Y88F-hDAT (*p* < 0.01), respectively. These results suggest that the mutations of Y470 and Y88 do not alter cocaine-induced competitive inhibition of DA uptake.

**Table 1:** Effects of SRI-20041 on cocaine-induced inhibition of [^3^H]DA uptake in WT and mutated hDAT.

Drug	WT-hDAT	Y470H-hDAT	Y470F-hDAT	Y88F-hDAT
	IC_50_ (nM)			
Cocaine	324 ± 19	196 ± 23^#^	281 ± 16	157 ± 10^#^
Cocaine + SRI-20041 (12.8 µM)	431 ± 32*	203 ± 28	394 ± 34*	156 ± 18
Cocaine + Indatraline (10 nM)	424 ± 23*	301 ± 38*	371 ± 32*	256 ± 31*

Data are presented as mean ± S.E.M. of 5–8 independent experiments performed in duplicate.

*p < 0.05 compared with the value of cocaine within WT or mutated hDAT (LSD post hoc test).

^#^ *p* < 0.05 compared with the value of cocaine from WT-hDAT (LSD post hoc test).

We next evaluated whether these mutations influence the effect of SRI-20041 on cocaine-induced inhibition of [^3^H]WIN35,428 binding site. As illustrated in Table 2, two-way ANOVA revealed that there were significant main effects of drug (F_(2,18)_ = 5.16, *p* < 0.05), mutation (F_(1,18)_ = 55.93, *p* < 0.001), and drug × mutation interaction (F_(2,18)_ = 6.95, *p* < 0.01). The IC_50_ value of cocaine in WT-hDAT (173 ± 26 nM) was decreased by 274% in Y88F-hDAT (63 ± 5 nM, *t*_(6)_ = 4.20, *p* < 0.01). The addition of SRI-20041 (12.8 but not 1.28 µM) produced a 64% decrease in the IC_50_ value of cocaine with a marginal significant effect (cocaine alone: 173 ± 26 nM; SRI-20041 + cocaine: 256 ± 18 nM, *p* = 0.058), indicating SRI-20041 dose-dependently alters cocaine-induced inhibition of [^3^H]WIN35,428 binding. However, SRI-20041 had no effect on the IC_50_ value of cocaine in Y88F-hDAT. Since the binding of [^3^H]WIN35,428 in Y470H-hDAT was dramatically decreased (~90%)^44^, the profile of [^3^H]WIN35,428 in Y470H-hDAT was not included in the current study.

**Table 2:** Effects of SRI-20041 on Cocaine IC_50_ of [^3^H]WIN 35,428 binding in WT and mutated hDATs.

Drug	WT-hDAT	Y470H-hDAT	Y88F-hDAT
	IC_50_ (nM)		
Cocaine	173 ± 26	N.D.	63 ± 5^#^
Cocaine + SRI-20041 (1.28 µM)	122 ± 35	N.D.	69 ± 6
Cocaine + SRI-20041 (12.8 µM)	256 ± 18*	N.D.	59 ± 4

Data are presented as mean ± S.E.M. of 4 independent experiments performed in duplicate.

*p = 0.05 compared with the value of cocaine within WT (LSD post hoc test).

^*#*^ *p* < 0.05 compared with the value of cocaine from WT-hDAT (Student’s unpaired *t* test).

N.D. indicated as non-detected.

### Mutations of Y470 and Y88 attenuate effects of SRI-20041 on cocaine-induced dissociation of [^3^H]WIN35,428 binding

**Figure 2:**
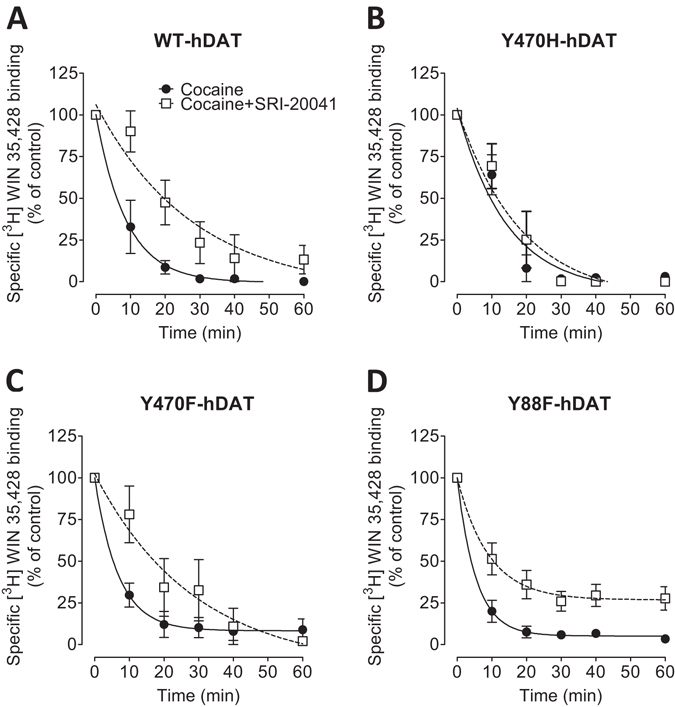
Effects of SRI-20041 and cocaine on the dissociation of [^3^H]WIN 35,428 in WT hDAT and mutants. PC12 cells transfected with WT hDAT (**A**) or Y470H-hDAT (**B**), Y470F-hDAT (**C**), and Y88F-hDAT (**D**) were incubated with [^3^H]WIN 35,428 (final concentration, 5 nM) on ice for 2 h. After the 2 h incubation, the incubation reagent was replaced with fresh assay buffer. At the zero time point, cocaine (1 µM) was added to Condition-1 and Condition-2 (also see Table 3). Ten minutes later, SRI-20041 (10 µM) was added to Condition-2. Cells were lysed at indicated time points. For the data analysis, the 100% control point (no drug) was time 0 for Condition-1 and time 10 min point for Condition-2. Each data point is the mean ± S.E.M. (*n* = 5–8).

The dissociation assay has been typically used for determining allosteric modulation of ligands^32, 37^. To verify the impact of Y470 and Y88 residues on SRI-20041-induced allosteric modulation of DAT function, the effects of SRI-20041 and cocaine on the dissociation of [^3^H]WIN35,428 binding were examined. As reported in Table 3 and Fig. 2, the dissociation of [^3^H]WIN35,428 binding with cocaine (1 µM) proceeded in a monotonic manner and was well described by a single component dissociation model in WT and its mutants, which is consistent with our previous report^35^. Goodness-of-fit analyses of individual experiments revealed R^2^s ranging from 0.988 ± 0.005 to 0.775 ± 0.056 among all experimental conditions (Supplementary Table 1a). Two-way ANOVA revealed that there were significant main effects of drug (F_(1,40)_ = 14.20, *p* < 0.01) and mutation (F_(2,18)_ = 6.30, *p* < 0.01). The dissociation rate in WT-hDAT (K_−1_ = 0.161 ± 0.032 min^−1^) was similar to that in Y470F-hDAT (K_−1_ = 0.161 ± 0.027 min^−1^) and not significantly changed in Y88F-hDAT (K_−1_ = 0.272 ± 0.060 min^−1^, LSD: *ps* > 0.05). Compared to WT, although Y470H-hDAT displayed a decrease in the dissociation rate, it did not reach the significant level (K_−1_ = 0.076 ± 0.007 min^−1^, *p* = 0.085). The addition of SRI-20041 following the addition of cocaine significantly slowed the dissociation rate in WT-hDAT (K_−1_ = 0.033 ± 0.009 min^−1^, *t*_(10)_ = 3.98, *p* < 0.01) and in Y470F (K_−1_ = 0.043 ± 0.024 min^−1^, *t*_(9)_ = 3.25, *p* < 0.01). However, the effect of SRI-20041 on cocaine-induced dissociation of [^3^H]WIN35,428 binding was attenuated in Y470H-hDAT (K_−1_ = 0.062 ± 0.019 min^−1^, *t*_(8)_ = 0.67, *p* = 0.52) and Y88F-hDAT (K_−1_ = 0.144 ± 0.030 min^−1^, *t*_(13)_ = 1.81, *p* = 0.09), respectively.

**Table 3:** Effects of SRI-20041 on cocaine-induced [^3^H]WIN 35,428 binding dissociation in WT and mutated hDAT.

Drug	WT-hDAT	Y470H-hDAT	Y470F-hDAT	Y88F-hDAT
	Dissociation rate (K_−1_, min^−1^)			
Condition 1 (cocaine alone)	0.161 ± 0.032	0.076 ± 0.007	0.161 ± 0.027	0.272 ± 0.060
Condition 2 (cocaine + SRI-20041)	0.033 ± 0.009*	0.062 ± 0.019	0.043 ± 0.024*	0.144 ± 0.030

Data are calculated as a percentage of [^3^H]WIN 35,428 binding dissociation rate in response to cocaine with or without SRI-20041 addition followed by nonlinear regression analyses using a single component dissociation model. Data are presented as mean ± S.E.M. of dissociation rate values from 5–8 independent experiments performed in duplicate. *p < 0.05 compared with the respective condition 1.

### Mutations of Y470 and Y88 attenuate effects of SRI-30827 on cocaine-induced inhibition of DA uptake

SRI-30827 has been recently characterized as another novel DAT allosteric modulator with a chemical structure similar to that of SRI-20041 but with higher potency for inhibition of DA uptake (0.5 nM) in rat synaptosomes^36^. We further determined the effect of SRI-30827 on cocaine-induced inhibition of [^3^H]DA uptake in WT and mutants. As shown in Table 4, there was a significant main effect of mutation (F_(2,25)_ = 49.01, *p* < 0.001). The cocaine-inhibited [^3^H]DA uptake with IC_50_ value (359 ± 30 nM) was significantly decreased in Y470H-hDAT (131 ± 35 nM, LSD: *p* < 0.001) and Y88F-hDAT (160 ± 27 nM, *p* < 0.001), respectively. The addition of SRI-30827 (50 nM) following addition of cocaine (1 µM) significantly increased the cocaine IC_50_ value to 458 ± 22 nM, *t*_(9)_ = 2.60, *p* < 0.05) in WT-hDAT. However, the allosteric modulatory effect of SRI-30827 on cocaine-mediated inhibition of DA uptake was attenuated in Y470H-hDAT (141 ± 35 nM) and Y88F-hDAT (161 ± 34 nM), respectively, relative to their respective controls (cocaine alone).

**Table 4:** Effects of SRI-30827 on cocaine-induced inhibition of [^3^H]DA uptake in WT and mutated hDAT.

Drug	WT-hDAT	Y470H-hDAT	Y88F-hDAT
	IC_50_ (nM)		
Cocaine	359 ± 30	131 ± 35^#^	160 ± 27^#^
Cocaine + SRI-30827 (50 nM)	458 ± 22*	141 ± 35	161 ± 34

Data are presented as mean ± S.E.M. of 5–6 independent experiments performed in duplicate.

*p < 0.05 compared with the value of cocaine within WT-hDAT (Student’s unpaired *t* test).

^#^ *p* < 0.05 compared with the value of cocaine from WT-hDAT (Student’s unpaired *t* test).

### Mutations of Y470 and Y88 attenuate effects of SRI-30827 on cocaine-induced dissociation of [^3^H]WIN35,428 binding

**Figure 3:**
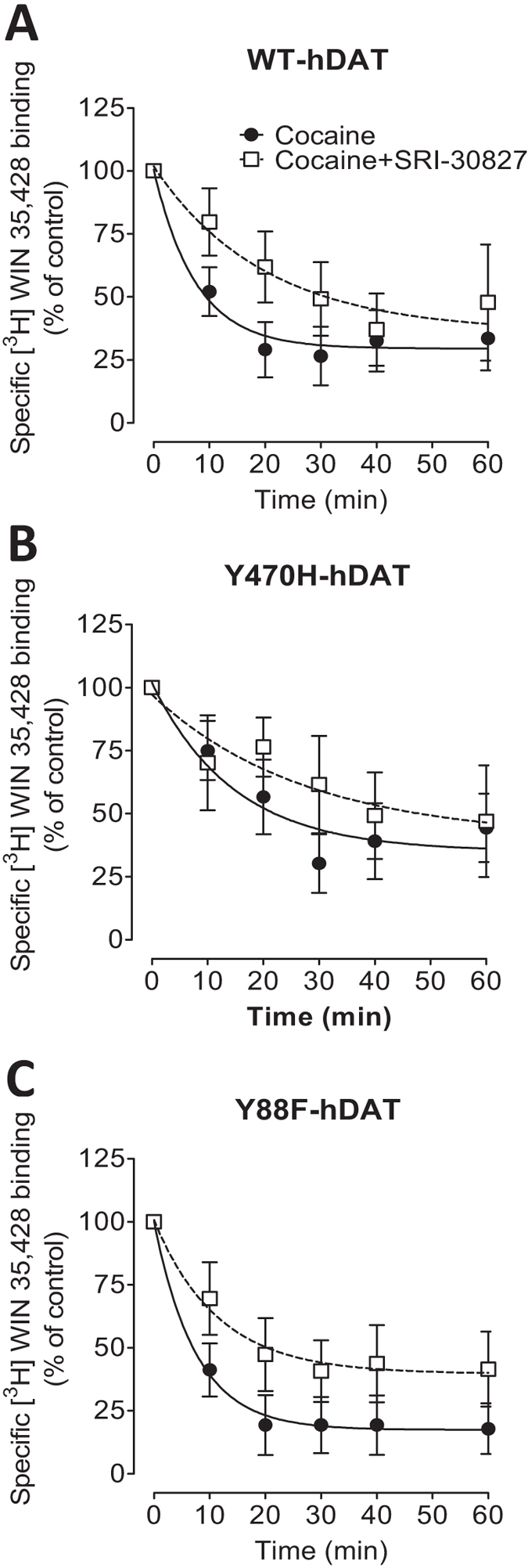
Effects of SRI-30827 and cocaine on the dissociation of [^3^H]WIN 35,428 in WT hDAT and mutants. PC12 cells transfected with WT hDAT (**A**) or Y470H-hDAT (**B**), and Y88F-hDAT (**C**) were incubated with [^3^H]WIN 35,428 (final concentration, 5 nM) on ice for 2 h. After the 2 h incubation, the incubation reagent was replaced with fresh assay buffer. At the zero time point, cocaine (1 µM) was added to Condition-1 and Condition-2 (also see Table 5). Ten minutes later, SRI-30827 (50 nM) was added to Condition-2. Cells were lysed at indicated time points. For the data analysis, the 100% control point (no drug) was time 0 for Condition-1 and time 10 min point for Condition-2. Each data point is the mean ± S.E.M. (*n* = 4–7).

As reported in Table 5 and Fig. 3, the dissociation of [^3^H]WIN35,428 binding with cocaine (1 µM) proceeded in a monotonic manner and was well described by a single component dissociation model in WT and its mutants (the range of R^2^: 0.975 ± 0.011 to 0.676 ± 0.159, Supplementary Table 1b). Two-way ANOVA revealed a significant main effect of drug (F_(1,26)_ = 8.57, *p* < 0.001) and a marginal main effect on mutation (F_(2,26)_ = 3.19, *p* = 0.058). The dissociation rate in WT-hDAT (K_−1_ = 0.148 ± 0.012 min^−1^) was not significantly changed in Y88F-hDAT (K_−1_ = 0.134 ± 0.018 min^−1^, LSD: *p* > 0.05). However, Y470H-hDAT displayed a decrease in the dissociation rate (K_−1_ = 0.068 ± 0.015 min^−1^, *p* < 0.001) compared to WT, indicating that mutation of Y470 alters cocaine-induced dissociation of [^3^H]WIN35,428 binding. The application of SRI-30827 following the addition of cocaine significantly slowed the dissociation of rate in WT-hDAT (K_−1_ = 0.054 ± 0.027 min^−1^, *t*_(11)_ = 6.46, *p* < 0.001). However, the effect of SRI-30827 on cocaine-induced dissociation of [^3^H]WIN35,428 binding was attenuated in Y470H-hDAT (K_−1_ = 0.061 ± 0.027 min^−1^, *t*_(7)_ = 0.23, *p* = 0.82) and Y88F-hDAT (K_−1_ = 0.095 ± 0.014 min^−1^, *t*_(8)_ = 1.69, *p* = 0.13), respectively, relative to their respective controls (cocaine alone).

**Table 5:** Effects of SRI-30827 on cocaine-induced [^3^H]WIN 35,428 binding dissociation in WT and mutated hDAT.

Drug	WT-hDAT	Y470H-hDAT	Y88F-hDAT
	Dissociation rate (K_−1_, min^−1^)		
Condition 1 (cocaine alone)	0.148 ± 0.012	0.068 ± 0.015^#^	0.134 ± 0.018
Condition 2 (cocaine + SRI-30827)	0.054 ± 0.027*	0.061 ± 0.027	0.095 ± 0.014

Data are calculated as a percentage of [^3^H]WIN 35,428 binding dissociation rate in response to cocaine with or without SRI-30827 (50 nM) addition followed by nonlinear regression analyses using a single component dissociation model. Data are presented as mean ± S.E.M. of dissociation rate values from 4–7 independent experiments performed in duplicate. *p < 0.05 compared with the respective condition 1 (Student’s unpaired *t* test). ^#^ *p* < 0.05 compared with WT-hDAT (LSD post hoc test).

### Effect of SRI-30827on Tat-induced inhibition of [^3^H]WIN35,428 binding in WT hDAT

**Figure 4:**
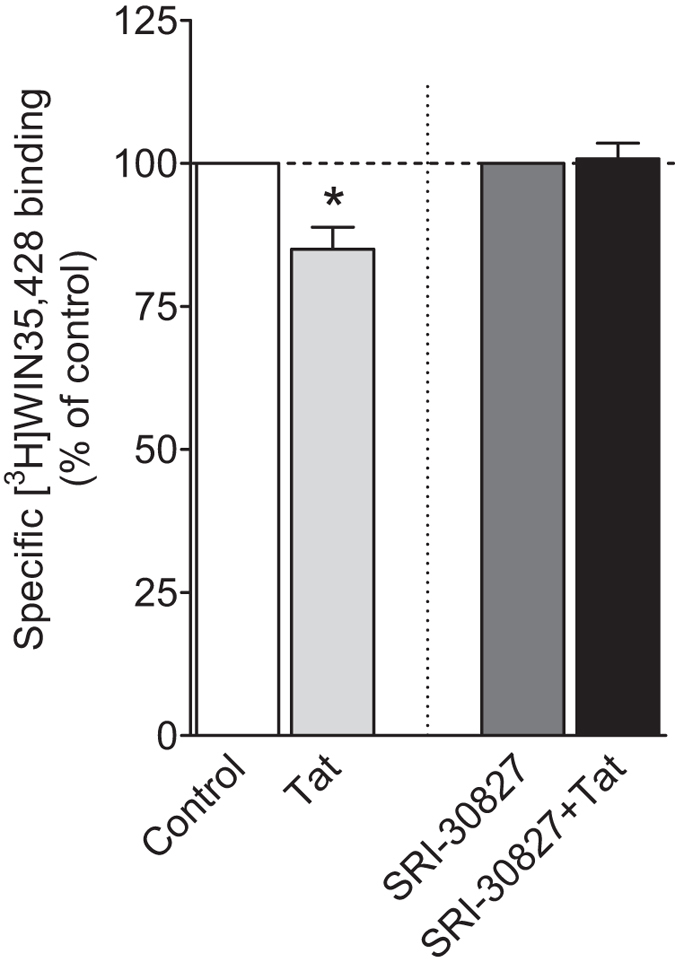
Effect of SRI-30827 (50 nM) on Tat-induced inhibition of [^3^H]WIN35.428 binding in WT hDAT. CHO cells transfected with WT hDAT were incubated with [^3^H]WIN 35,428 (final concentration, 5 nM) with or without recombinant Tat_1–86_ (rTat_1–86_) (40 nM, final concentration) on ice for 2 h. Nonspecific uptake was determined in the presence of 30 µM final concentration of cocaine. Data for rTat_1–86_ and SRI-30827 + rTat_1–86_ are expressed as the percentage of their respective controls in the absence of rTat_1–86_ (15675 ± 2982 DPM) and SRI-30827 (14041 ± 3212 DPM) n = 5. *p < 0.05 compared with control value.

We have demonstrated that Tat protein produces inhibitory effects on DAT function and expression. To determine whether the Tat-induced inhibition of DAT binding sites is mediated through an allosteric modulation manner, the ability of SRI-30827 to attenuate the inhibitory effect of Tat was determined (Fig. 4).

Two-way ANOVA revealed that the main effect of Tat on the specific [^3^H]WIN35,428 binding was significant (F_(1,20)_ = 7.62, *p* < 0.05), whereas the main effect of SRI-30827 was not significant (F_(1,20)_ = 3.42, *p* = 0.08). Moreover, a significant SRI-30827 × Tat interaction (F_(1,20)_ = 4.08, *p* < 0.05) was observed. Compared with control, Tat (40 nM) induced a decrease (14 ± 3.3%) in the specific [^3^H]WIN35,428 binding in WT hDAT (t_(5)_ = 4.2, *p* < 0.01, unpaired Student’s *t* test). SRI-30827 itself did not alter the specific [^3^H]WIN35,428 binding (*p* > 0.05); however, SRI-30827/Tat showed no inhibitory effect on the specific [^3^H]WIN35,428 binding compared to SRI-30827 alone, indicating that SRI-30827 attenuates Tat-induced decrease in DAT binding sites.

## Discussion

The current study reports the allosteric modulatory effects of SRI-compounds on cocaine and Tat binding to hDAT. This study is an extension of our previous work demonstrating that mutations of Y470 and Y88 of hDAT are critical for Tat-induced inhibition of DA transport and conformational transporter transitions^44, 47, 50^. There are three major findings from the current study. First, using indatraline to assess competitive DAT inhibition, we found that the SRI-compounds and indatraline produce a similar increase in the IC_50_ value of inhibition of [^3^H]DA uptake by cocaine in WT hDAT; however, the SRI-compounds-induced change in the IC_50_ of cocaine is attenuated in Y470H and Y88F. Second, using SRI-compounds to assess allosteric modulation of DAT function, the addition of either SRI-20041 or SRI-30827 following the addition of cocaine significantly slowed the dissociation rate of [^3^H]WIN35,428 binding compared to cocaine alone, which was totally and partially blocked in Y470H and Y88F, respectively. Third, SRI-30827 attenuates Tat-induced inhibition of [^3^H]WIN35,827 binding in WT hDAT. The significance of the current observations is two-fold: 1) this is the first report on the key residues responsible for the allosteric modulatory effects on cocaine and Tat binding to hDAT, and 2) the Y470 and Y88 may be related to the allosteric modulatory effects for the SRI-compounds and the Tat binding to hDAT. These findings provide a novel mechanistic basis for developing compounds that specifically attenuate cocaine and Tat binding site(s) in hDAT to normalize DA transmission to physiological levels in HIV-infected cocaine-using patients.

HIV-1 Tat protein and cocaine synergistically disrupt normal physiological dopaminergic transmission by inhibiting DAT. Attenuating inhibitory effect of Tat and cocaine on DA transport is important for preventing the DAT-mediated dysfunction of DA system in HIV infected patients with cocaine abuse. Cocaine binding sites are overlapped with DA uptake sites^31^, which makes it impossible to generate a competitive inhibitor of cocaine binding that treats cocaine addiction without itself inhibiting DA uptake. However, if inhibition of DA uptake by cocaine is the result of an allosteric mechanism, it would be possible, at least in theory, to generate an allosteric modulator for treatment of cocaine addiction that might attenuate cocaine binding without affecting DA transport. In general, drugs that interact with DAT are typically classified into two categories: 1) inhibitors, such as cocaine and GBR12909, and 2) substrates, such as DA and amphetamine. However, there is growing interest in allosteric modulation of DAT. Conformational transitions via substrate- and ligand-binding sites on DAT are responsible for allosteric modulation of DAT^55^. The conformational changes in the DA transport process involve three known conformational states of hDAT (occluded, outward-open and inward-open)^31, 56, 57^. Cocaine preferentially stabilizes the DAT in the outward-open state, resulting in reduction of DA uptake by directly blocking DA uptake site^58, 59^. Thus, characterization of the binding sites in DAT responsible for cocaine-induced conformational changes will provide a mechanistic basis to identify targets on the DAT for developing novel compounds that reduce cocaine’s potency via their allosteric modulation of DA transport process. The present study shows that SRI-20041 and SRI-30827 allosterically modulate inhibition of [^3^H]DA uptake by cocaine. Moreover, DAT dissociation assays show that the addition of SRI-20041 or SRI-30827 after cocaine significantly slows the dissociation rate. This is consistent with previous reports from our laboratory^35^ and by others^32, 36^, suggesting that these two SRI-compounds modulate cocaine binding to DAT through an allosteric modulation mechanism. Notably, the previous studies were conducted in rat striatal synaptosomes while the current study is the first to determine the allosteric modulatory effects of SRI-compounds in PC12 cells expressing hDAT. Given the important role of DAT in addictive effects of cocaine^29, 30^, determining the effects of SRI-compounds in cocaine-mediated behavior change is an interesting topic for future investigations.

The most intriguing observation is that mutants Y470H and Y88F do not appear to interact with cocaine but lead to an attenuation of allosteric modulation of SRI-compounds on cocaine-mediated inhibition of DA transport and cocaine-induced dissociation of [^3^H]WIN35,428 binding. Our computational modeling predicts that both cocaine and Tat interact with hDAT in the outward-open conformational state, resulting in a synergistic inhibitory effect on DA transport^50^. Interestingly, Y470H and Y88F displayed a higher efficacy in inhibiting DA uptake and DAT binding by cocaine compared with WT hDAT^44, 47^. In addition, the previous results show that Y470H- and Y88F-mutations altered zinc-induced regulation of DA uptake and WIN35,438 binding^44, 47^. One possibility is that Y470H- and Y88F-mediated transporter conformational transitions may contribute to these changes in cocaine-mediated inhibition of DA uptake and dissociation in these mutants. Earlier studies have shown that Phenylalanine472 and Leucine475 that are in proximity of Y88 and Y470 are critical for the interaction of cocaine with DAT^31, 60^, indicating a complex interaction of cocaine and Tat with DAT. The complex interaction of cocaine and Tat may change the sensitivity to cocaine. This may contribute to the clinical and preclinical observations showing an acceleration of HAND in HIV-infected patients who abuse cocaine^8, 9, 13^ and the neurocognitive/behavioral deficits observed in HIV-1 transgenic animals^49, 61^. Therefore, these results suggest that Y470H and Y88F play a critical role in the synergistic effects of cocaine and Tat on DAT.

This study provides novel mechanistic insight that Y470 and Y88 residues may influence the allosteric modulation sites on hDAT for both SRI-20041 and SRI-30827. We have demonstrated that Tat-induced inhibition of DAT is mediated by allosteric binding site(s) on DAT, not the interaction with the DA uptake site^35, 45, 50^. Tat molecule binds to DAT through intermolecular electrostatic attractions and complementary hydrophobic interactions^50^. In particular, eliminating the cation-π interaction between Y470 in hDAT and M1 in Tat by mutating Y470 into a residue such as histidine or alanine would significantly weaken the binding between hDAT and Tat^47^. Further, in light of the Tat-DAT binding structure, mutation of Y88 to a residue without hydrogen-bonding capacity would also weaken the Tat-DAT binding^47^. These computational predictions are validated by experimental data showing that Tat-induced inhibition of DAT is attenuated in Y470H and Y88F^44, 47^. Furthermore, Y470H but not Y470F attenuates Tat-induced inhibition on DAT because mutating Y470 to phenylalanine (Y470F) does not affect the cation-π interaction^47^. In the current study, we also found that SRI-compounds-induced decrease in cocaine IC_50_ was attenuated in Y470H but not in Y470F, further supporting the important role of Y470 in the Tat-DAT interaction. By using a hDAT homology model to dock Tat into the transporter and MD simulations to probe the conformational state of hDAT bound to Tat, we found that Tat can only bind to the outward-open structure with favorable binding energies^50^. Therefore, we predicted that Tat binding would block the entry pathway of the dopamine substrate, thereby inhibiting dopamine clearance from the presynaptic cleft. Since the conformational changes in DA transport process involve conversions between the outward- and inward-open conformations^57^, we then conducted docking studies using homology models of hDAT at the different conformational states and found that the two SRI-compounds could only fit into the outward-open hDAT model (Fig. 1). Further, both SRI-20041 and SRI-30827 interact with DAT extracellular loop 6 (EL6) that contacts directly with Tat and can partially inhibit DAT uptake function with a similar chemical structure^36^. According to our previous modeling results, in the outward-open hDAT conformation, Y470 extends to the extracellular region where it interacts directly with Tat residues^50^. In the SRI-compound docked models, SRI-compounds may potentially influence the conformation of residues Tyr470 and Tyr88 with EL6 region, and thus likely modulate the binding of Tat on hDAT via an allosteric modulatory mechanism. Hence, although SRI-compounds may not interact directly with either Tyr470 or Tyr88 for competing with Tat binding, they can weaken the Tat DAT binding by changing the DAT conformation allosterically. This has been verified by our experimental data showing that SRI-30827 attenuates Tat-induced inhibition of DAT binding sites. Taken together, considering both Y470 and Y88 are associated with hDAT-Tat interactions, developing compounds directly targeting the specific binding sites on hDAT for Tat could be a viable approach for treatment of Tat-induced dopamine dysfunction. Alternatively, developing DAT-based allosteric modulators interacting with the specific residues that are structurally distinct from Tat binding sites would be another possible therapeutic approach.

In conclusion, we have identified that Y470 and Y88 may act as allosteric modulatory sites on hDAT responsible for the effects of cocaine binding displayed by the two allosteric ligands, SRI-20041 and SRI-30827. Given that mutation of these two residues (Y470H and Y88F) attenuates Tat-induced inhibition of DAT function^44, 47^, our current study also demonstrates an allosteric modulatory effect of SRI-30827 on attenuation of Tat-induced inhibition of DAT binding sites. These findings presented herein raise the exciting possibility of potential therapeutic intervention for HIV infected patients with concurrent cocaine abuse. Proof of this concept could emerge from efforts directed toward discovery and development of candidate *in vivo* probe molecules with the desired allosteric modulation profiles coupled with favorable drug-like attributes. The effectiveness of an early intervention for HAND to preserve neurocognitive functions in HIV-infected individuals may ultimately depend on a treatment approach that combines compound(s) that specifically attenuate Tat binding site(s) in DAT with antiretroviral therapy, without affecting the normal function of DAT.

## Methods

### Materials

PC 12 cells (ATCC^®^ CRL-1721^TM^) were obtained from ATCC (Manassas, VA). 3,4-[7-^3^H]DA (28 Ci/mmol) and [N-methyl-^3^H]WIN 35,428 (82.62 Ci/mmol) were purchased from Perkin Elmer (Boston, MA). The novel DAT allosteric modulators, SRI-20041 and SRI-30827, were synthesized at Southern Research Institute (Birmingham, AL). Cocaine hydrochloride and other fine chemicals/reagents were from Sigma-Aldrich (St. Louis, MO) unless otherwise noted.

### Molecular Modeling

As shown in Fig. 1, molecular docking was performed to determine the structures of hDAT binding with SRI-compounds by using the AutoDock Vina^62^ based on the constructed hDAT-Tat complex from our previous work^50^. Both the substrate-binding site and extracellular region of hDAT were defined as the search space for the docking. For each SRI-compound, 20 independent docking processes were performed in order to explore all possible binding conformations in such a large search space. The best protein-ligand binding conformation for each SRI-compound was selected according to the best binding score given by the AutoDock Vina. Further, the atomic charges for SRI-compound molecule were determined as restrained electrostatic potential (RESP)-fitted charges based on the first-principles electronic structure calculations at the B3LYP/6-31 G* level by using the Gaussian03 program^63^. For each SRI-compound, the selected binding conformation was combined with the constructed hDAT-Tat structure with tLeap of the AMBER12 package^64^. Then the SRI-hDAT-Tat complex was optimized with 100,000 steps of the deepest decent energy-minimization and 100,000 steps of the conjugate gradient energy-minimization by using the Sander module of the AMBER 12 package^64^.

### Construction of plasmids

Point mutations of hDAT at Y470 (tyrosine to histidine, Y470H-hDAT; tyrosine to phenylalanine, Y470F-hDAT) and Y88 (tyrosine to phenylalanine, Y88F-hDAT) were selected based on the structural and mechanistic insights obtained from computational modeling and simulation and previous studies^47, 50^. The Y470H-hDAT is expected to eliminate both hydrogen bond and cation-π interactions in the native structure of the transporter. Both Y470F-hDAT and Y88F-hDAT are expected to preclude the formation of hydrogen bond. All mutations were generated according to WT-hDAT sequence (NCBI, cDNA clone MGC: 164608, IMAGE: 40146999) by QuikChange^TM^ site-directed mutagenesis kit (Agilent Tech, Santa Clara, CA) with synthetic cDNA encoding hDAT subcloned into pcDNA3.1 + (a gift provided by Dr. Haley E Melikian, University of Massachusetts) as the template. The sequences of mutated constructs were further confirmed through DNA sequencing at University of South Carolina EnGenCore facility. Plasmid DNA were propagated and purified using a plasmid isolation kit (Qiagen, Valencia, CA).

### Cell culture and DNA transfection

PC12 cells were maintained in the incubator (37 °C, 5% CO_2_) with DMEM medium supplemented with horse serum (15%) and bovine calf serum (2.5%), glutamine (2 mM), and penicillin-streptomycin (100 U/ml). Chinese hamster ovary cells (CHO cells, ATCC, CRL-61) were maintained in F12 medium with 10% fetal bovine serum and antibiotics (100 U/mL penicillin and 100 µg/mL streptomycin). For hDATs transfection, cells were seeded into poly-D-lysine-coated 24 well plates at a density of 1 × 10^5^ cells/well. After 24 h, cells were transfected with WT or mutant DAT plasmids using Lipofectamine 2000 (Life Tech, Carlsbad, CA) based on the manufacturer’s instruction. Cells were used for the experiments after 24 h of transfection.

### Competitive inhibition [^3^H]DA uptake assay

Considering that PC12 cells endogenously expressing norepinephrine transporter (NET) and DA is transported by DAT and NET, in a pilot study, we first performed kinetic analysis of [^3^H] DA uptake in PC12 cells with no transfection in the presence of desipramine (1 µM, final concentration) to prevent DA uptake into norepinephrine containing nerve terminals based on a previously published method^65^. Results showed that no specific [^3^H] DA uptake was found with a range of DA concentrations (1 nM-5 µM, data not shown). [^3^H] DA uptake assay in PC12 cells transfected with WT or mutated hDATs was performed 24 hr after transfection based on our previous studies^35, 44^. Briefly, assays were performed in duplicate in a final volume of 500 µl. Cells were washed twice with Krebs-Ringer-HEPES (KRH) buffer (concentration in mM: 125 NaCl, 5 KCl, 1.5 MgSO_4_, 1.25 CaCl_2_, 1.5 KH_2_PO_4_, 10 D-glucose, 25 HEPES, 0.1 EDTA, 0.1 pargyline, and 0.1 L-ascorbic acid; pH 7.4). After washing, cells in each well were incubated with KRH buffer with one of ten concentrations of cocaine (1 nM-1 mM) and a fixed concentration of SRI-20041 (12.8 µM), SRI-30827 (50 nM) or indatraline (10 nM) for 10 min at room temperature. [^3^H]DA (final concentration: 0.1 µM) was added for additional 8 min. In parallel, the non-specific [^3^H]DA uptake was determined in the presence of nomifensine (10 µM). The doses of SRI-compounds and indatraline were based on previous publications^32, 35^. Uptake reaction was terminated by three more washes of ice-cold KRH buffer. Cells were then lysed in 1% SDS and the remaining [^3^H]DA radioactivity in each well was counted by liquid scintillation spectrometry.

### [^3^H]WIN 35,428 binding assay

The competitive binding experiments were performed in duplicate in a final volume of 250 µl. Twenty four hours after transfection with WT or mutant hDATs, PC12 cells were washed once with sucrose-phosphate assay buffer (concentration in mM: 2.1 NaH_2_PO_4_, 7.3 Na_2_HPO_4_7H_2_O, and 320 sucrose). Cells were then incubated with one of eleven concentrations of cocaine (1 nM-1 mM) and fixed concentrations of SRI-20041 (12.8 µM or 1.28 µM) or SRI-30827 (50 nM) in the presence of [^3^H]WIN 35,428 (5 nM) on ice for 2 hr in duplicate. After incubation, the intact cells in each well were washed twice with ice-cold assay buffer, lysed by 1% SDS for 1 hr and subjected to liquid scintillation spectrometry measurement. For the binding dissociation assay, experiments were conducted in a final volume of 500 µl according to our previous publication^35^. The intact cells were incubated with a fixed concentration of [^3^H]WIN 35,428 (5 nM) on ice for 2 hr followed by a wash of ice-cold assay buffer. Non-specific [^3^H]WIN 35,428 binding was determined by addition of β-CFT naphthalenedisulfonate monohydrate (1 µM). In condition 1 (cocaine only), at the zero time point, the binding dissociation was initiated by the application of a single concentration of cocaine (1 µM). In condition 2 (cocaine + SRI-compounds), 10 min after the addition of cocaine, SRI-20041 (10 µM) or SRI-30827 (50 nM) was then added to minimize any [^3^H]WIN35,428 re-association. Ten, 20, 30, 40, and 60 min later, duplicate wells of cells were washed twice with assay buffer, lysed, and counted by liquid scintillation spectrometry. For data analyses, 0 min (no drugs treatment) and 10 min after the application of cocaine were set as 100% to normalize samples at each time point in condition 1 and 2, respectively, based on previous publications^32, 35^.

Our previous study shows that Tat inhibits [^3^H]WIN35,428 binding, which is consistent with the ability of Tat inhibiting DA uptake^45^. To determine the effect of SRI-30827 on Tat-induced inhibition of DAT binding sites, the specific [^3^H]WIN35,824 binding (5 nM, final concentration) in WT hDAT was measured in the presence or absence of SRI-30827 (50 nM, final concentration) or recombinant Tat_1–86_ (40 nM, final concentration, ImmunoDX, Woburn, MA) on ice for 2 h. This concentration of SRI-30827 was chosen because our pilot study showing SRI-30827 at this concentration did not alter basal [^3^H]WIN35,824 binding and also this concentration was used for the dissociation assay. Nonspecific [^3^H]WIN35,824 binding was determined in the presence of 30 µM cocaine.

### Data analyses and Statistics

Descriptive statistics and graphical analyses were used as appropriate. Results are presented as mean ± SEM, and *n* represents the number of independent experiments for each experiment group. IC_50_ values for cocaine inhibition of [^3^H]DA uptake and [^3^H]WIN35,428 binding were calculated from inhibition curves by nonlinear regression analysis with a one-site model of variable slope. For the dissociation of [^3^H]WIN35,428 binding induced by cocaine, the dissociation rate (K_−1_) was determined by the specific [^3^H]WIN35,428 binding through non-nonlinear regression analysis using a single component dissociation model. Both kinetic parameters (cocaine IC_50_ and K_−1_) were calculated by Garphad Prism version 5.0 (GarphPad Software Inc., San Diego, CA). For experiments involving comparisons between unpaired samples, separate ANOVAs followed by appropriate post hoc tests [Fisher’s least significant difference (LSD) or Student’s unpaired *t* test] were used. All statistical analyses were performed using IBM SPSS Statistics version 24. *p* < 0.05 was the minimum criterion for statistical significance.

## Electronic supplementary material

Supplementary Information
