# Supplementary material for: Allosteric modulatory effects of SRI-20041 and SRI-30827 on cocaine and HIV-1 Tat protein binding to human dopamine transporter
Source: Sci Rep. 2017 Jun 16;7:3694. doi: 10.1038/s41598-017-03771-0 (PMC5473888; doi:10.1038/s41598-017-03771-0)
Supplement: Supplementary file 1 — Supplementary Information [file 41598_2017_3771_MOESM1_ESM.pdf]

Supplemental Table 1a Summary of coefficient of determination (R^2) in goodness-of-fit analyses of one-site model of [^{3}H]WIN 38,428 binding dissociation in WT-hDAT and mutated hDAT in the presence of cocaine and SRI-20041

Drug	WT-hDAT	Y470H-hDAT	Y470F-hDAT	Y88F-hDAT
	R^2			
Condition 1 (cocaine alone)	0.948 ± 0.041	0.901 ± 0.044	0.983 ± 0.007	0.988 ± 0.005
Condition 2 (cocaine +SRI-20041)	0.775 ± 0.056	0.915 ± 0.026	0.911 ± 0.035	0.914 ± 0.18

Supplemental Table 1b Summary of coefficient of determination (R^2) in goodness-of-fit analyses of one-site model of [^{3}H]WIN 38,428 binding dissociation in WT-hDAT and mutated hDAT in the presence of cocaine and SRI-30827

Drug	WT-hDAT	Y470H-hDAT	Y88F-hDAT
		R^2	
Condition 1 (cocaine alone)	0.892 ± 0.062	0.794 ± 0.079	0.975 ± 0.011
Condition 2 (cocaine +SRI-30827)	0.828 ± 0.058	0.676 ± 0.159	0.885 ± 0.056
